# Chemically-induced trout model of acute intestinal inflammation using TNBS

**DOI:** 10.1016/j.fsirep.2022.100073

**Published:** 2022-11-29

**Authors:** Marianna E. Horn, Helmut Segner, Markus Brinkmann, Steven Machtaler

**Affiliations:** a Department of Medical Imaging, University of Saskatchewan, 103 Hospital Drive, Saskatoon, Saskatchewan S7N 0W8, Canada; b Centre of Fish and Wildlife Health, University of Bern, Länggassstrasse 122, 3012 Bern, Switzerland; c School of Environment and Sustainability, Toxicology Centre, and Global Institute for Water Security, University of Saskatchewan, 44 Campus Drive, Saskatoon, Saskatchewan S7N 5B4, Canada

**Keywords:** rainbow trout, intestinal inflammation, enteritis, TNBS, chemically-induced inflammation

## Abstract

Chemically-induced models of intestinal inflammation are a useful tool for the study of immune responses and inflammation. Although well established in mammals, application of these models is currently limited in teleosts. Based on a variety of factors, including genetic diversity, known toxicological sensitivity, and economic importance, we propose salmonids as a model family of fishes for studying intestinal inflammation. We present a rainbow trout model of chemically-induced intestinal inflammation using 2,4,6-trinitrobenzene sulfonic acid (TNBS), assessed through histological analysis of primary and secondary intestinal folding, enterocyte morphology, goblet cell size and frequency, tissue layer thickness, and immune cell infiltration. Twenty-four hours after treatment with one of three concentrations of TNBS, trout developed classic signs of intestinal inflammation, including notably increased thickness of primary and secondary folds, and increased immune cell infiltration as compared to controls. This study provides a simple, reproducible model of rapid TNBS-induction of moderate intestinal inflammation.

## Highlights

• Study of pro-inflammatory potential of 2,4,6-trinitrobenzene sulfonic acid (TNBS) in fish
• Rectal administration of TNBS induces intestinal inflammation in rainbow trout
• Intestines inflamed using TNBS exhibit increased thickness and immune cell infiltration

## 1 Introduction

Models of intestinal inflammation in mammals have been well-established using mice, rats, and rabbits [[1], [2], [3]] and have led to a fundamental understanding of how the adaptive and innate immune systems initiate inflammation and disease in the intestine [[4], [5], [6]]. Various chemical induction models of intestinal inflammation exist that can initiate an innate-mediated immune response through oral administration of dextran sulfate sodium (DSS) or an adaptive T-cell mediated immune response through a localized, intrarectal administration of 2,4,6-trinitro benzene sulfonic acid (TNBS) or oxazolone [7,8].

Administration of TNBS in mammals results in a T-cell mediated intestinal inflammatory response localized to the region of contact within the intestine [1,9]. Acting as a haptenizing agent, the TNBS penetrates the ethanol-permeabilized intestinal mucosal epithelium and becomes antigenic after binding to autologous proteins. This drives an immune response, particularly in mammalian CD4+ T-cells [9]. The damage induced by TNBS administration is clinically relevant for models of both ulcerative colitis and Crohn's disease, making it a versatile choice for the general study of acute gastrointestinal inflammation found in inflammatory bowel diseases [10].

Teleosts have both immunological and intestinal systems that show evolutionary parallels to those of mammals. They have both innate and adaptive immune systems, including cells functionally equivalent to T- and B-cells, and similar structures and functions of intestinal architecture [[11], [12], [13], [14]]. Various studies have explored induction of intestinal inflammation in zebrafish with TNBS, DSS, or oxazolone via either intrarectal administration or larval immersion [[15], [16], [17], [18], [19]]. As in mammals, administration of TNBS in adult zebrafish results in an inflammatory response, with increases in pro-inflammatory cytokines including tumor necrosis factor alpha (TNF-α) [16], as well as alterations of the intestinal morphology including an increase of leukocytes and goblet cells [17].

Models of intestinal inflammation have great potential for understanding inflammatory changes in teleosts, but these models have not been broadly adapted beyond zebrafish. Zebrafish are a common choice because of the availability of genetic information and the relative ease of genetic manipulation. However, there are limitations in restricting teleost studies of intestinal inflammation to zebrafish. Their small size and short lifespan make them ideal for the lab, but extensive breeding may also bias the genetics of the model. Beyond this, different clades of fish are known to have varied sensitivities to toxins [20,21], and therefore establishing models within different phylogenetic groups is important. A repeatable method of initiating robust and spatially restricted intestinal inflammation in various clades of teleosts can give insights into immune responses, be compared to different materials that induce inflammation, such as novel feeds, and can be used as a method to test anti-inflammatory therapies.

Salmonids are considered both environmentally important, as a key element in many ecosystems, and economically valuable, as they are prized for game fishing and heavily cultivated for aquaculture. They are widespread, found across North America, Europe, and Asia. This environmental ubiquity makes them an excellent model for studying the role of toxicants in inflammatory responses within the intestines. Rainbow trout (*Oncorhynchus mykiss*), a member of the salmonid family, represent an advantageous model for the study of intestinal inflammation. Trout are ecologically naturalized over a wide range and are genetically more diverse than the extensively inbred zebrafish [22,23]. They are physically larger and have longer lifespans. They are also economically important as both cultured food and game fish [24]. Here we describe a rainbow trout model of intestinal inflammation. We show that intestinal inflammation can be initiated using rectal administration of TNBS and is characterized by changes to the lamina propria and immune cell infiltration. This ecologically relevant model provides enormous potential for investigating numerous factors impacting intestinal health, including environmental pollutants and toxins, as well as effects of different types of feeds in aquaculture settings.

## 2 Methods

### 2.1 Induction of acute TNBS colitis

#### 2.1.1 Holding conditions

Triploid female rainbow trout eggs were acquired from Lyndon Fish Hatcheries (Petersburg, ON, Canada) and were raised in the University of Saskatchewan's Aquatic Toxicology Research Facility for approximately two years. Prior to our experiment they were held in 900-L circular flow-through tanks at 14°C equipped with aeration and biofilters and were fed commercial dry pellets (Corey Aquafeeds 3 mm floating trout pellets, Corey Nutrition Company, Fredericton, Canada) *ad libitum* once daily. All trout were of the same age and developmental stage.

#### 2.1.2 Treatment doses

To evaluate the efficacy of TNBS to induce intestinal inflammation, fifteen subadult trout (mean weight ± SD: 260 ± 72 g) were subjected to one of five treatments (n=3). There was no difference in weight between treatment groups (ANOVA: F_4,14_ = 1.57, P = 0.256). Three concentrations of TNBS were prepared: 0.8%, 1.3%, 2.5% wt/vol (g / 100 ml) in a 50:50 solution of saline and 95% ethanol. Two controls were prepared: saline (500 µl), and ethanol (50:50 solution of saline and 95% ethanol).

#### 2.1.3 TNBS Administration

**Fig. 1:**
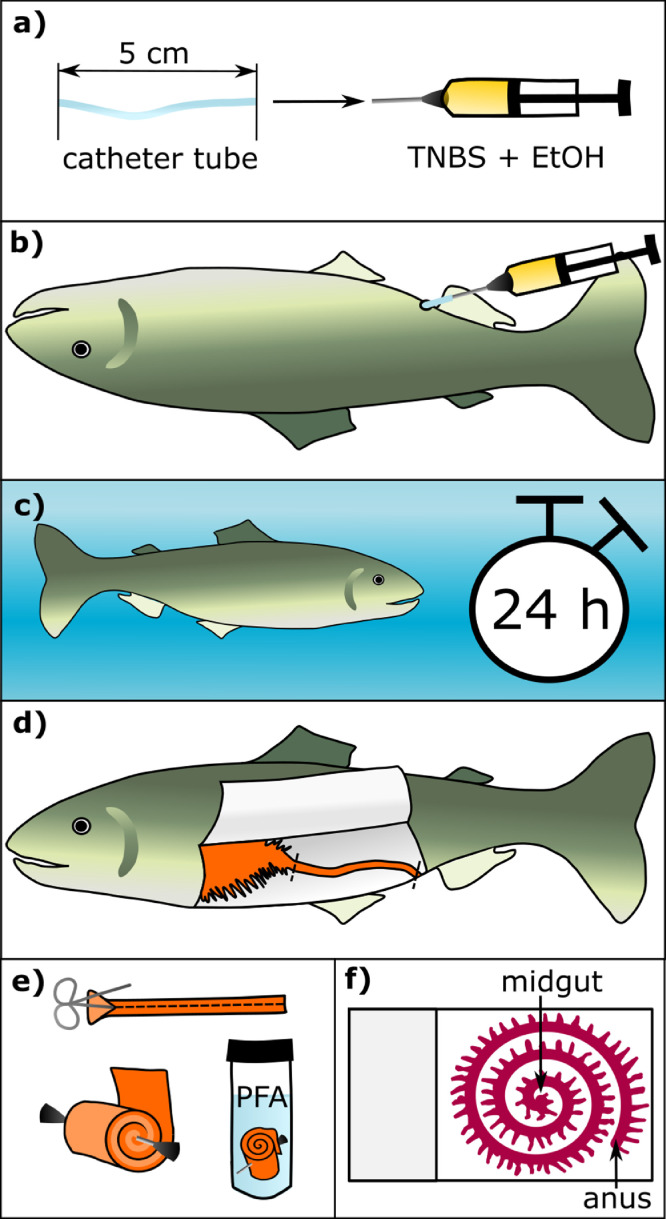
Schematic of TNBS administration procedure and tissue processing. a) A syringe was inserted into a plastic catheter line. b) Treatments were administered rectally in an anaesthetized trout. c) The trout was returned to a holding tank for 24 h. d) The trout was euthanized, and the intestines were collected via the midline. e) The intestine was split, rolled into a “Swiss roll,” and placed in paraformaldehyde. f) Histological slides show the full length of the intestinal section.

Two days prior to treatment, the trout were transferred to rectangular 700-L raceway tanks and fasted for 48 hours. On the day of treatment, each trout was placed in a bucket with an anaesthetic dose of buffered MS-222 (tricaine methanesulfonate, 75 mg / L; sodium bicarbonate 100 mg / L) [25] to minimize both distress and risk of inoculation trauma. Each trout was weighed and marked with a small fin clip to allow identification. A 5 cm length of 1.0 mm catheter tubing was fixed to an 18-gauge needle on a 1 ml syringe, and a 500 µL treatment dose was drawn up (Fig. 1a). Using very gentle pressure, we slid gloved fingers along the abdomen of the trout below the pectoral fins towards the anus to encourage expulsion of any residual fecal matter. The trout was held on its back in a shallow tray of MS-222-dosed anaesthetic water, keeping the gills wet and the anus above the water, positioning the tray at an angle as necessary. The catheter tube was lightly lubricated with ultrasound gel before gently inserting approximately 4 cm into the intestine via the anus (Fig. 1b). The catheter tubing was held approximately parallel to the body, and care was taken not to apply pressure that might damage or perforate the intestine wall during insertion. The treatment dose was slowly injected into the intestine, and the catheter tubing was withdrawn after a pause of approximately 20 s, intended to reduce the likelihood of immediate expulsion of the TNBS solution. The trout were then returned to their holding tank and carefully monitored for signs of distress for 24 hours (Fig. 1c). The 24 h incubation period was selected based on murine models. We needed to allow adequate time for inflammation to occur but preferred a shorter period for ethical reasons (to minimize potential distress). We anticipate that using varied incubation periods may optimize investigation of different points in the inflammatory pathway.

### 2.2 *Ex vivo* analysis of intestinal tissue

#### 2.2.1 Tissue preparation

Twenty-four hours after treatment, the trout were euthanized by MS-222 overdose (150 mg / L with sodium bicarbonate 150 mg / L) followed by severance of the spinal cord. The 5 cm segment of intestine closest to the anus was harvested immediately via a ventral midline incision using scissors and forceps (Fig. 1d). Intestines were opened using a longitudinal incision, flattened, and immediately dipped in a 4% paraformaldehyde (PFA) solution in phosphate-buffered saline (PBS) to prevent autolysis. The intestines were then rolled into a tight “Swiss roll” [26,27], with the mucosa facing outward and the most distal end (nearest the anus) outmost on the roll (Fig. 1e). This roll provides histological images which represent the entire length of the section, rather than a cross-section at a specific point. The roll was pinned and fixed overnight at 4°C in the 4% PFA/PBS solution. The intestine was then inserted in histocassettes and processed in 70% ethanol at 4°C for three 24-h periods, each with fresh ethanol. Tissues were then processed using a Leica Pearl Automated Tissue Processor set on a standard setting for muscle tissue before embedding in paraffin. Five µm cross-sections were produced using a microtome and adhered to microscopic slides. The sections were stained using standard hematoxylin-eosin (H&E) staining, with 1.5 minutes of immersion in the hematoxylin (Fig. 1f). Bright-field microscopy was used to produce images for histological evaluation.

#### 2.2.2 Histological evaluation

Evaluation of the microscopic images taken from each of the fifteen fish was first completed blind by our histologist, who first designated each sample as inflamed or not. We then verified whether these designations matched the TNBS versus control treatments and followed this with a detailed observation of the inflammatory indicators present in the different treatments. We specifically elected not to perform quantitative histology because of the extreme complexity of preparing intestinal samples with the precision necessary to provide to quantify. Given the variance that can result from even slight imprecisions, we believe that a qualitative evaluation provides the most accurate representation of our findings.

In mammalian intestines, the ratio of villi to crypts of Lieberkühn is considered a hallmark of inflammatory changes. However, because the intestinal folds of fish intestines are not equivalent to the villi of the mammalian intestine and fish intestines lack crypts of Lieberkühn, this ratio cannot be used. The diagnostic value of the fold morphology in fish is therefore limited. To this end, our histological analysis considers a variety of parameters, including the extent of primary and secondary intestinal folding, enterocyte morphology, goblet cell size and frequency, tissue layer thickness, and the presence of immune cells. Distinct intestinal segments or regions are not well defined in trout, apart from some changes in fold height and secondary folding, but the focus of the histological analysis was on the second segment of the mid-intestine [28]. References to the anterior, middle and posterior will describe relative location within the collected section.

### 2.3 Ethical Note

All research reported was conducted in accordance with the University of Saskatchewan Animal Care Committee under protocol number 20210022 and used a total of 15 sub-adult rainbow trout. The trout were subjected to fasting for three days. MS-222 was administered before the treatments. The trout were carefully monitored after administration, and none exhibited signs of distress during the 24 hours permitted for the chemicals to take effect. The trout were euthanized with buffered MS-222 followed by severance of the spinal column, per university animal care guidelines.

## 3 Results

### 3.1 General response and gross morphology

**Fig. 2:**
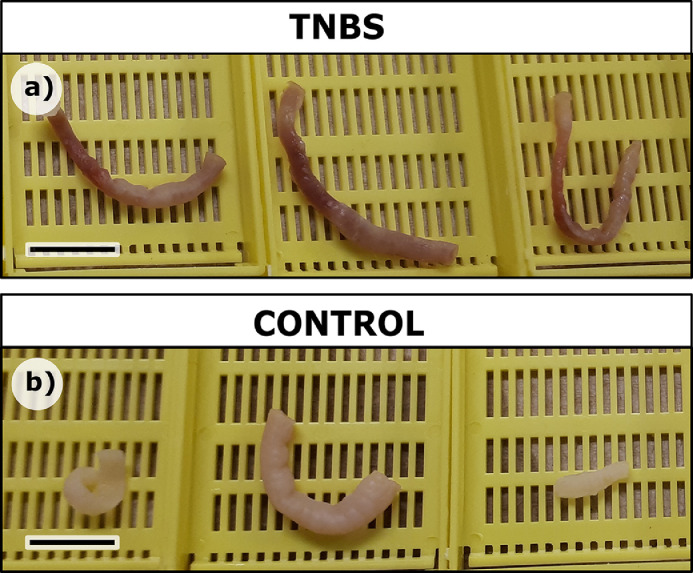
Gross morphology of bowels treated with TNBS or control. a) 2.5% TNBS treatment, b) saline treatment. Scale bar 1 cm.

Twenty-four hours after administration, there was no mortality and there were no overt behavioural changes, with fish from all treatments swimming freely in their raceway tank. After PFA fixation, the intestines treated with 2.5% TNBS appeared visibly reddened (Fig. 2a) compared to the saline-treated control intestines (Fig. 2b). Blind histological evaluation of microscopic slides accurately designated the nine fish treated with TNBS as inflamed, and the six control fish (both saline and ethanol) as uninflamed.

### 3.2 Histopathology of control trout intestines

**Fig. 3:**
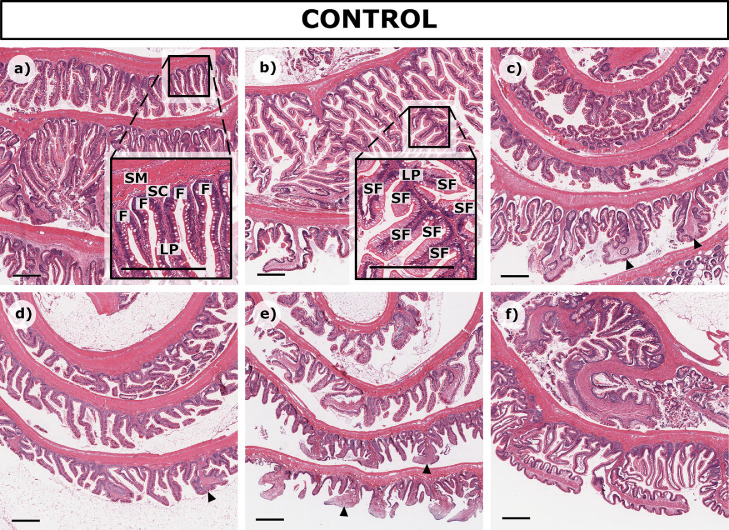
Normal mucosal folding in rainbow trout intestine. The extent of mucosal folding and secondary folding varies both between individuals and along the length of the intestinal region, as exhibited in six control fish: a) Saline Trout 1, b) Saline Trout 2, c) Saline Trout 3 d) Ethanol Trout 1, e) Ethanol Trout 2, f) Ethanol Trout 3. Inset highlights a) folding **F**, and b) secondary folding **SF** in relation to the lamina propria **LP**, the stratum compactum **SC** and the submucosa **SM**; triangles ▲ indicate flattening of the folds. Scale bar = 500 µm.

Control trout exhibited histological characteristics consistent with healthy intestines, with the mucosa of the intestinal wall arranged in irregularly shaped and randomly oriented folds (Fig. 3a, inset). The folds were highest and had the highest number of secondary folds in the middle region of the intestine (Fig. 3b, inset), with a trend of fold height decreasing towards the anterior or posterior regions. In the posterior region, the tips of some intestinal folds were flattened (Fig. 3, triangles ▲) and the lamina propria was generally broader. The extent of mucosal folding varied between individuals (Fig. 3) as is typical in mammals.

**Fig. 4:**
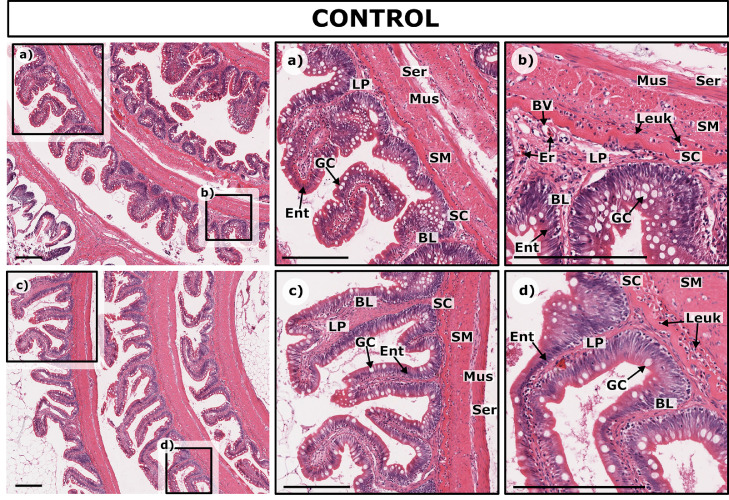
Normal mucosal and submucosal histology in control rainbow trout. Details of intestinal mucosa are seen in a) Saline Trout 3, c) Ethanol Trout 1 and d) Ethanol Trout 1, with folds composed of the epithelium (made up of enterocytes **Ent** and goblet cells **GC**) resting on the basal lamina **BL** and surrounding the lamina propria **LP**. Beneath this lies the stratum compactum **SC**, the submucosa **SM** and muscularis **Mus,** and the thin layer of the serosa **Ser**. Additionally, blood vessels **BV**, erythrocytes **Er,** and a few leukocytes **Leuk** are seen in b) Saline 3 and. Scale bar = 250 µm.

The intestinal wall was composed of mucosa, submucosa, muscularis and serosa (Fig. 4a-c). The mucosa consisted of a simple columnar epithelial layer, containing primarily enterocytes and goblet cells, which rested on a basal lamina. The enterocytes mostly displayed a basophilic apical cytoplasm, with some exceptions as, for instance, in parts of the intestinal epithelium in certain fish (Fig. 4b, d), where the apical cytoplasm of the enterocytes was filled with light vacuoles.

The epithelium contained a high number of goblet cells, with some regional variation (the highest goblet cell density was in the middle region, as in Fig. 4 a,b,d). In the center and at the base of the intestinal folds, there was loosely arranged connective tissue, the lamina propria (Fig. 4), which contained blood vessels and diverse cell types including fibrocytes and leukocytic infiltrates. Underlying the mucosa, there was the submucosa with a prominent stratum compactum, which contained a strong collagen sheath, blood vessels, and immune cells including granulocytes. (Fig. 4). To the serosal side, the next layer was the muscularis, with an inner circular and an outer longitudinal layer of smooth muscles, all of which were covered by a thin serosa (Fig. 4). Mononuclear leukocytes, primarily lymphocytes but also macrophages, were dispersed in the lamina propria and submucosa. Both submucosa and muscularis were relatively thin, with no consistent discrepancies between the various intestinal regions.

### 3.3 Histopathology of TNBS-treated trout intestines

Overall, congruent histological alterations demonstrating intestinal inflammation were observed with all three doses of TNBS (0.8, 1.3 and 2.5%). Though interindividual variations were pronounced, the severity of changes generally increased with the TNBS dose. Moreover, an increased TNBS dose appeared to correlate with the extension of the lesions spatially along the intestine rather than with an enhanced severity of pathology. The following features of intestinal morphology were evaluated for changes in TNBS-treated fish compared to control fish:

#### 3.3.1 Intestinal folds

**Fig. 5:**
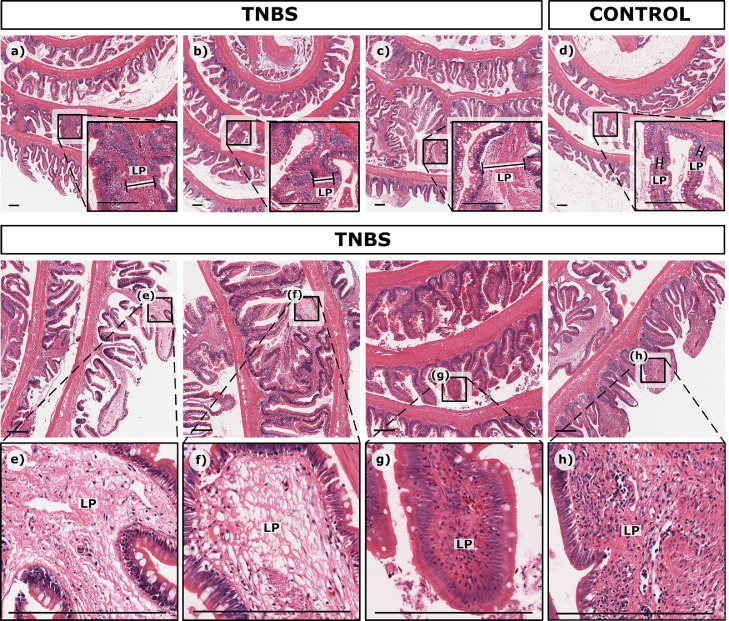
Histological samples highlighting intestinal folding in rainbow trout treated with different concentrations of TNBS. Intestinal folding was thicker and more had more secondary folds in fish treated with TNBS, a) 0.8% TNBS Trout 2, b) 1.3% TNBS Trout 3, c) 2.5% TNBS Trout 1, as compared to control d) Ethanol Trout 1, as a result of widening of the lamina propria **LP**, highlighted in the insets. The lamina propria widened either as loose filling shown in e) 2.5% TNBS Trout 3 and f) 2.5% TNBS Trout 1, or dense filling shown in g) 2.5% TNBS Trout 2 and h) 0.8% TNBS Trout 1. Scale bar = 250 µm.

**Fig. 6:**
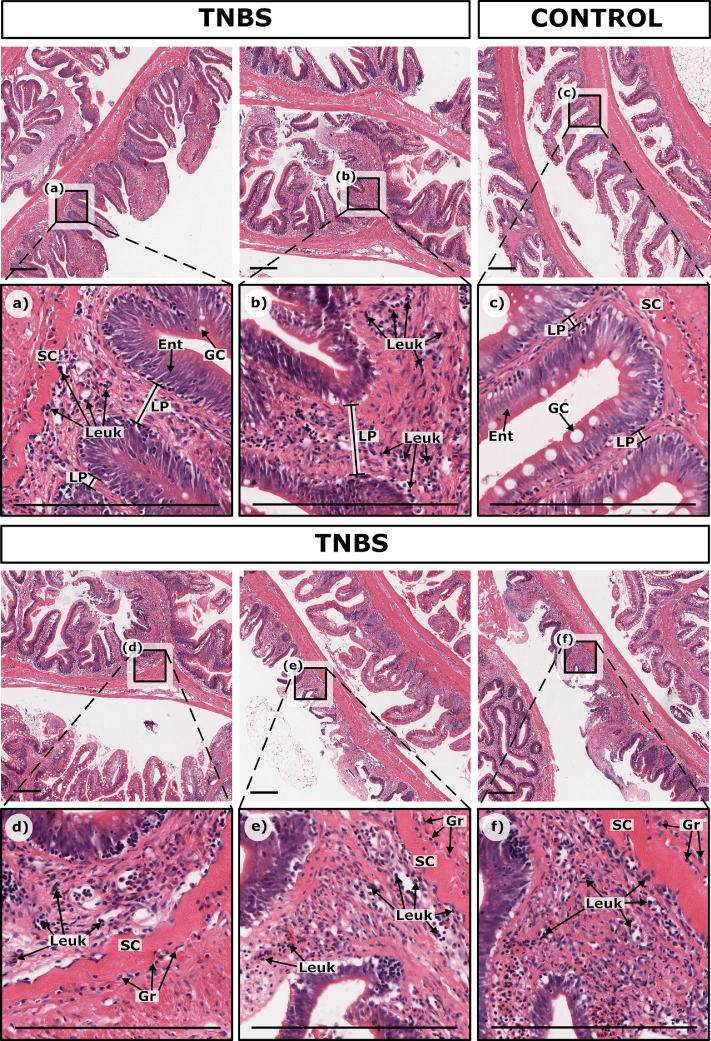
Thickening of the lamina propria and increased immune cell infiltration in TNBS treated rainbow trout. In treatment with TNBS, the lamina propria **LP** was thickened, and space between the stratum compactum **SC** and mucosal fold base was enlarged and infiltrated by leukocytes **Leuk** as in a) 0.8% TNBS Trout 1 and b) 2.5% TNBS Trout 1, in contrast with the smaller space in control treatments seen in c) Ethanol Trout 1. In some cases, intestines treated with TNBS also exhibited an increased number of granulocytes **Gr** along the stratum compactum as in d) 2.5% TNBS Trout 1, e) 1.3% TNBS Trout 3 and f) 2.5% TNBS Trout 1.

The intestinal folds in TBNS-treated trout were thicker and had formed more secondary folds than in control trout (Fig. 5a-d). The increased fold thickness was due to an increase in lamina propria width (Fig. 5a-d and Fig. 6a-c). Secondary folding and thickening of the mucosa folds were expressed most severely in the middle region of the intestine (Fig. 5a-d). The lamina propria structure could be loose (Fig. 5e-f) or dense (Fig. 5g-h), displaying connective fibres as well as invading leukocytes and increased congestion. No decrease in fold height was evident in this study.

#### 3.3.2 Enterocyte morphology

The morphology of the enterocytes did not differ between treated and control (Fig. 6 a-c). The apical cytoplasm was partly filled with absorptive vacuoles but varied among individuals and between intestinal regions and was not related to treatment.

#### 3.3.3 Thickness of tissue layers

The widths of the overall submucosa, stratum compactum, and muscularis displayed no apparent increase with TBNS treatment (Fig. 6 a-f). In contrast, the thickness of the mucosal layer was partly increased due to the widening of the lamina propria.

#### 3.3.4 Presence of immune cells

The most characteristic and significant change after TNBS administration was an increased number of leukocytes particularly in the lamina propria and partly in the submucosa (Fig. 6). Both lymphocytes, most prominent in the lamina propria, and granulocytes, particularly in the region around the stratum compactum, were present. In the 0.8% TNBS treatment, an increase in leukocyte numbers was not apparent, with numbers remaining at a similar level to the control trout. However, with increasing TNBS dose, an increasing leukocyte infiltration became visible. The ratio of lymphocytes and granulocytes varied between individuals; for instance, though one trout with a 2.5% TNBS treatment might have had a prominent granulocyte response, lymphocytes might have dominated in another with the same treatment. Along with the leukocyte infiltration, the lamina propria widened, as explained above, and the space between stratum compactum and the base of the intestinal folds increased in size (Fig. 6a-c) which should be considered when assessing the number of leukocytes.

#### 3.3.5 Goblet cells

**Fig. 7:**
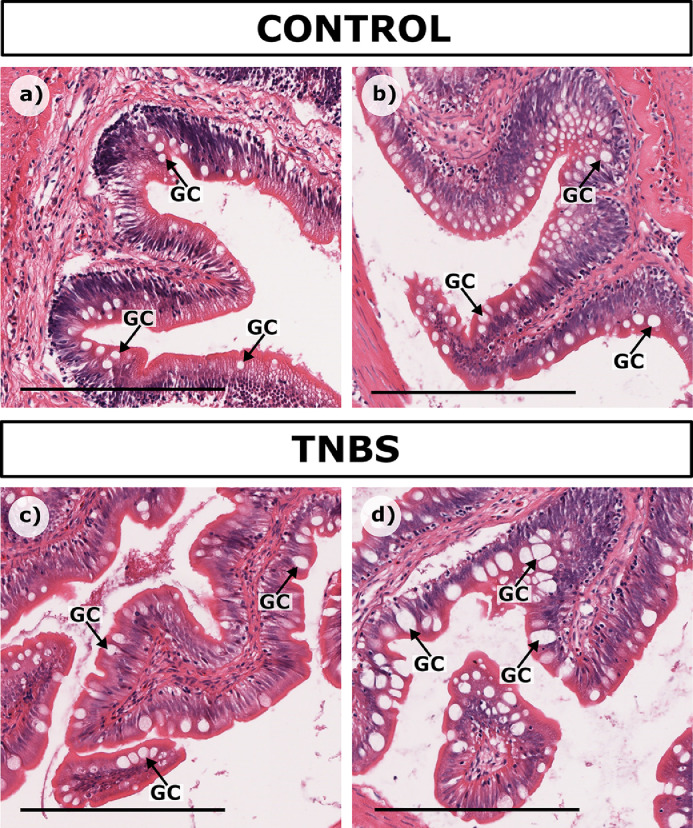
Variance in size and abundance of goblet cells between control and TNBS-treated rainbow trout. Compared to controls a) Control Trout 2 and b) Ethanol Trout 1, goblet cells in the higher TNBS treatments appeared to increase in size as seen in c) 1.3% TNBS Trout 3, d) 2.5% TNBS Trout 1. Scale bar = 250 µm.

The goblet cells showed no change in frequency but appeared to increase in size in the 1.3 and 2.5 % TNBS groups (Fig. 7). In these groups, an increased percentage of the goblet cells appeared to be releasing mucus into the gut lumen, but alternative histological stains would have been required to verify this [14].

Overall, the inflammation in the trout intestines treated with TNBS displayed distinct enteritis (inflammation) characterized by increased thickness and secondary folding of the intestinal mucosa, a trend of increased size of goblet cells in the higher two concentrations, and increased immune cell infiltration compared with controls, with the intensity gradually increasing with TNBS dose.

## 4 Discussion

We present a rainbow trout model of gastrointestinal inflammation in which we observed an increase in immune cell presence, thickening of the lamina propria and increased secondary folding 24 h after administration of a single dose of TNBS without need for presensitization. In contrast to the intestinal folds, thickening did not occur in the tissue layers below, including the submucosa, stratum compactum, or muscularis. The morphology of the enterocytes and the numbers of goblet cells did not change.

Few studies have explored the pathomorphological effects of intrarectal injection of TNBS into teleost intestines. Ji et al. [29] provided the first description of this type of administration in salmonids in a study in which they explored the protective effects of β-glucan against TNBS-induced inflammation, however this study provides limited detail on the histology, which is a key technique for overall assessment of intestinal inflammation. Other studies with in-depth histological analyses of responses to intrarectal administration of TNBS focused exclusively on zebrafish. These studies consistently observed both immune cell infiltration and thickening of the intestinal folds, as seen in our study, across a variety of dosages and incubation periods [17,30]. Similar thickening of the folds was also observed with administration of oxazolone in place of TNBS [15]. Whole-body larval immersion also consistently resulted in increased numbers of immune cells but varied in changes to the intestinal folds [16,31]. No changes were noted to the number of goblet cells in adult zebrafish exposed to intrarectal TNBS administration [17], which parallels our observations. Although this is in contrast to an increase in the number of goblet cells observed in TNBS-immersed larvae [16], goblet cell variations do not appear to be consistent and therefore are not considered a key parameter of intestinal inflammation, as opposed to leukocyte infiltration and changes in the lamina propria. The histological findings in teleosts also parallel the infiltration by immune cells observed in mammalian systems [1,32].

This study provides a technique for inducing moderate intestinal inflammation in trout. Repeatable models such as the one we present are essential to our understanding of inflammatory processes, including the biochemical and immunological pathways that lead to these responses. In addition, they allow for the development of tools to study inflammatory responses, such as novel techniques to evaluate the degree of inflammation in fish intestines. Zebrafish are the primary teleost model for bowel inflammation and offer a lot of potential for studies, including genetics and omics. However, even among teleosts, there is significant variation in diet and consequently in morphological and chemical digestive adaptations, such as the simple intestinal bulb found in zebrafish [11] in contrast with the glandular stomach found in rainbow trout [33]. These and other physiological variations make it important to study and compare different clades of fishes, and development of this model of inflammation in trout affords an excellent opportunity to compare biological variability.

Heavy metals, pesticides, pharmaceuticals, and other pollutants may all affect intestinal inflammatory responses, and the model of inflammation we have established in rainbow trout can be used as a baseline for comparison with these and other toxicants. Additionally, trout are known to suffer from nutritionally-induced intestinal inflammation, which can impact growth and general health and is therefore of economic concern in aquaculture. For example, many species of fish have strong inflammatory responses to feed containing soy products [[34], [35], [36]]. Indeed, decreases in fold height have been reported in some cases of nutritionally-induced enteritis of fish [37]. Our trout model can be used as a positive control when considering nutritionally-induced inflammation, and can be used to develop tools to improve evaluation of intestinal inflammation.

Although this study presents a model of intestinal inflammation in rainbow trout, it is not without limitations. Our model describes acute inflammation but does not represent the complex biology of chronically inflamed tissues, which merit further exploration. Although we used three dosages of TNBS, we did not explore the differences between the responses to these doses quantitatively. This model was intended to determine at what dose an inflammatory response was initiated, and we found that our 2.5% TNBS dose worked well without mortality or serious complications. This is important, as using the same technique, at dosages about four times greater than ours (per g of body mass) are reported to incur mortality rates up to 50% in zebrafish [17]. Future studies may investigate differences between doses (for example, using 0.8% as mild and 2.5% as moderate) to explore changes in the inflammatory response or may study chronic inflammation to compare with this acute inflammatory model.

## 5 Conclusion

We have developed a reproducible TNBS model of intestinal inflammation in rainbow trout marked by thickening of the mucosal folds and immune cell infiltration. Our model provides a technique for rapid induction of acute intestinal inflammation in an ecologically applicable species which can be used as a positive control for comparison with a wide variety of inflammatory agents.

## Author Contributions

This experiment was conceptualized by M.H., M.B., and S.M.. Experiments were executed by M.H. Analysis of histological images was performed by H.S. The original draft was prepared by M.H.. All authors contributed to the final manuscript.

## Declaration of Competing Interest

The authors declare that they have no known competing financial interests or personal relationships that could have appeared to influence the work reported in this paper.

## Data Availability

No data was used for the research described in the article.
